# Recommendations for the use of vascular access in the COVID-19 patients: an Italian perspective

**DOI:** 10.1186/s13054-020-02997-1

**Published:** 2020-05-28

**Authors:** Mauro Pittiruti, Fulvio Pinelli, on behalf of the GAVeCeLT Working Group for Vascular Access in COVID-19, Mauro Pittiruti, Fulvio Pinelli, Maria Giuseppina Annetta, Sergio Bertoglio, Daniele G. Biasucci, Roberto Biffi, Simona Biondi, Fabrizio Brescia, Massimo Buononato, Giuseppe Capozzoli, Paolo Cotogni, Elisa Deganello, Laura Dolcetti, Daniele Elisei, Stefano Elli, Davide Giustivi, Emanuele Iacobone, Antonio La Greca, Massimo Lamperti, Giada Maspero, Giancarlo Scoppettuolo, Davide Vailati, Daniele Vezzali

**Affiliations:** 1 Department of Surgery, Catholic University Hospital, Largo Gemelli 8, 00168 Rome, Italy; 2 Department of Anesthesia and Intensive Care, Careggi University Hospital, Florence, Italy

**Keywords:** COVID-19, Vascular access devices, Central venous catheters

## Background

During the COVID-19 pandemic, a group of experts from the Italian association GAVeCeLT (*Gruppo Accessi Venosi Centrali a Lungo Termine*) has formulated a few recommendations for the selection, insertion, and maintenance of the venous access devices, designed to protect the operator, to ensure the effectiveness of the maneuver, to reduce the risk of complications, and to save resources.

1. Recommendations on the appropriate choice of peripheral venous access:
  In the COVID-19 patient who does not require admission to intensive care unit (ICU), use a peripheral venous access device; prefer a long peripheral cannula (a.k.a. “mini-Midline,” 6–15 cm) [1] or a standard midline catheter (15–25 cm) rather than a short cannula (< 6 cm). Due to their longer dwell time, midline catheters will reduce the number of peripheral venous insertions required (thus saving resources and reducing risks for the operator); they will allow high flow infusions and easy blood sampling; if indicated, they might be easily replaced over guidewire with a peripherally inserted central catheter (PICC).
2. Recommendations on the appropriate choice of central venous access:
  In the COVID-19 patient in ICU, use a central venous access: PICC, FICC (femorally inserted central catheter), or CICC (centrally inserted central catheter).
  Consider the advantages of using PICCs in patients with severe respiratory distress (no risk of pleuropulmonary complications at insertion), in pronated patients (easy management of the exit site), in anticoagulated patients (no risk of bleeding), in patients on CPAP/NIV (little or no interference with the respiratory management), and/or in tracheostomized patients (lower risk of contamination of the exit site and of exposure of the operator to the patient’s tracheal secretions, if compared to CICCs). Power injectable PICCs can be used safely in ICU [2–4]: they are not associated with additional thrombotic risk if compared to CICC [5, 6], and they are appropriate for measurement of central venous pressure [7] and of cardiac output by thermodilution [8, 9].
  Consider the use of femoral access (FICCs) so to minimize the risk of operator contamination by the patient’s oral, nasal, and tracheal secretions during insertion. Choose an exit site at mid-thigh, away from the groin, either by puncturing the common femoral vein and then tunneling to mid-thigh or by directly puncturing the superficial femoral vein at mid-thigh.
  When inserting a CICC, prefer an infraclavicular approach (ultrasound-guided puncture and cannulation of the axillary vein) rather than a supraclavicular approach, so to provide greater protection and stability of the catheter at the exit site.
  In the absence of contraindications, give low molecular weight heparin at prophylactic (100 units/kg/24 h) or even therapeutic (100 units/kg/12 h or 150 units/kg/24 h) dose in all COVID-19 patients with central lines, so to reduce the thrombotic risk.
3. Recommendations on the appropriate choice of insertion technique:
  Use ultrasound guidance for the insertion of any central venous access or midline catheter or peripheral arterial catheter [10–13].
  Prefer wireless ultrasound probes, as they allow maximal cleaning of the probe between patients and minimal risk of contamination. In the unavailability of wireless ultrasound probes, the best strategy is to dedicate an ultrasound device exclusively to maneuver on COVID-19 patients.
  Avoid radiology after central venous cannulation: either transporting the patient to the radiology suite or bringing the radiological equipment to the patient’s bed, the risk of contamination of operators and machinery is very high. The location of the tip of the central venous catheter can be verified by non-radiological methods, such as transthoracic echocardiography (TTE) and intracavitary electrocardiography (IC-ECG) [13, 14]. Tip location by TTE is performed rapidly at bedside using wireless probes with convex or sectorial transducers and adopting the so-called bubble test (rapid infusion of saline with the addition of micro-bubbles of air, visualized by subxiphoid or apical echocardiography) [15]. Tip location by IC-ECG can be easily performed at bedside, either with a standard ECG monitor or with a dedicated wireless ECG monitor.
  After CICC insertion, immediately after the venipuncture, verify the absence of pleural damage by ultrasound examination of the pleural space, which is more accurate than chest x-ray in the diagnosis of pneumothorax [11, 12].
  As the risk of central venous catheter dislodgment is particularly high in the COVID-19 patient, particularly during the maneuvers of pronation-supination, consider the use of subcutaneously anchored securement.
  Protect the exit site of the catheter sealing it with cyanoacrylate glue (so to prevent local bleeding) and covering it with semi-permeable transparent membranes.
4. Recommendations on the appropriate precautions to avoid operator contamination:
  For patient protection, adopt the standard barrier precautions (hand hygiene, skin antisepsis with 2% chlorhexidine in 70% isopropyl alcohol, non-sterile surgical mask, non-sterile cap, sterile gloves, waterproof sterile gown, wide sterile field on the patient, sterile probe cover of appropriate length).
  For protection of the operator, adopt the standard personal protective equipment for contact protection (double glove, full suit, goggles or face shield, footwear); use both a surgical mask and a protective mask with N95 filter (equivalent to FFP2 of the European nomenclature), considering the high risk of aerosol in the environment, especially in the extubated and symptomatic COVID patient on NIV.

## Conclusions

The COVID-19 pandemic will undoubtedly change many of our clinical behaviors in the future. We hope that in the field of venous accesses, the positive side effect of this experience can take the form of a new awareness of the need to save resources and increase safety even outside of health emergency situations, adopting winning strategies such as the following:

- To implement vascular access teams trained to insert any short- or medium-term venous access device, according to the needs of the individual patient.
- To abandon the routine use of radiology for checking the tip location and ruling out pneumothorax after central venous access insertion, in favor of faster, more accurate, safer, and cheaper methods such as intracavitary electrocardiography and echocardiography;
- To adopt systematically appropriate technique of infection prevention in order to maximize both patient and operator safety during insertion of vascular access devices.

## Data Availability

Not applicable
